# The Relationship between the Frontal QRS-T Angle on ECG and Physical Activity Level in Young Adults

**DOI:** 10.3390/ijerph20032411

**Published:** 2023-01-29

**Authors:** Constantin Ciucurel, Elena Ioana Iconaru

**Affiliations:** Department of Medical Assistance and Physical Therapy, University of Pitesti, 110040 Pitesti, Romania

**Keywords:** electrocardiography, frontal QRS-T angle, physical activity regime, body mass index, young people

## Abstract

Background: The heart’s electrical activity has been the subject of numerous research concerning various physiological parameters. The frontal QRS-T angle (FQRST) is an advanced ECG variable with clinical epidemiological utility. This study aimed to determine the relationship between FQRST and physical activity exposure among young adults. Methods: We recorded the ECG with 12 leads of 124 participants (mean age 20.28 ± 2.23 years, age range 18–27 years). Next, we measured their physical activity level (PAL) with the International Physical Activity Questionnaire—Short Form (IPAQ), which categorizes activity into three classes: low, moderate, or high. Results: An inferential analysis, based on the Kruskal-Wallis H test and Mann–Whitney U test, revealed a statistically significant difference in FQRST between the three groups of subjects, classified by their PAL (*p* < 0.001). We also identified a significant regression model between the body mass index (BMI) and the FQRST (*p* < 0.001). Conclusions: The physical activity regime of young adults significantly influences the concordance between ventricular depolarization and repolarization, reflected in the FQRST’s width. Also, we found a regression model between FQRST and BMI with statistical significance.

## 1. Introduction

### 1.1. The Frontal QRS-T Angle on ECG in Epidemiological Studies

The heart’s electrical activity has been the subject of numerous research concerning various physiological parameters. The frontal QRS-T angle (FQRST) is an advanced ECG variable with clinical epidemiological utility, especially for long-time prognosis of cardiac mortality and risk in the general population [1,2]. The difference in orientation between ventricular depolarization and repolarization can be analyzed as the size of the FQRST. This angle is delimited between the mean QRS and T wave vectors. In the frontal plane, the mean frontal QRS axis (FQRSA) reflects the main direction of ventricular depolarization, while the mean frontal T axis (FTA) corresponds to the main direction of ventricular repolarization [3]. In conventional ECG with 12 leads, the FQRST angle is calculated as the absolute value of the difference between the FQRSA and FTA. If this difference is higher than 180°, the final FQRST represents 360° minus the absolute value of FQRST [4]. Most ECG devices automatically determine the FQRSA and FTA but not the FQRST. A simple additional algebraic calculation is required for the determination of FQRST. These variables could also be calculated manually, and the results are similar to or even better than in the automated version. Thus, in some cases, manual measurements are necessary to correct the ECG data resulting from machine measurement [2].

The FQRST has been proposed in clinical practice for the prediction of many other variables, but there are still controversies regarding the interpretation of research results [5]. This parameter is not routinely used since advanced knowledge is required for its determination and analysis. Recent studies have demonstrated that FQRST enlargement is associated with the aging process [6], the risk of cardiac arrhythmias, such as atrial fibrillation [7], hypertension [8], the severity of coronary artery disease and myocardial infarction [9,10], heart failure [11], idiopathic dilated cardiomyopathy [12], etc. It is also recommended for stratifying cardiac risk [13] and for monitoring patients after cardiac resynchronization therapy [14], myocardial revascularization, or cardiac valve surgery [15].

Several authors reported differences in FQRST depending on sex, with women having a smaller angle at baseline than men [13]. However, others did not find such evidence in the middle-aged general population [1]. Nomograms have also been proposed to interpret the FQRST values according to age and sex. The deviations from these references have a prevalence of approximately 2% in the general population [16].

### 1.2. The Relationship between Physical Activity and ECG Parameters

ECG changes in the context of regular physical training are common and usually reflect a physiological adaptation of the heart. The experts have systematized the main changes in the heart’s electrical activity induced by exercise. Thus, the common training-related (physiological) ECG markers are as follows: increased QRS amplitude (left or right ventricular hypertrophy), ST-segment elevation, incomplete right bundle branch block, sinus bradycardia, physiological arrhythmias, and early repolarization/ST-segment elevation, ectopic atrial or junctional rhythm [17,18].

The regular and long-term intense training regimes (minimum of 4 h per week) determine particular ECG changes as a consequence of electrical and morphological adaptations secondary to repeated sessions of exercise [18,19]. Generally, these adaptations consist of heart remodeling, cardiac cavities enlargement, and increased vagal tone [18,19]. Although sometimes pathological or borderline changes may appear, most of the time, the ECG findings in physically active people are considered normal and do not require supplementary evaluation or intervention [18].

As borderline ECG changes in athletes, we note the left or right axis deviation, voltage criteria for left or right atrial enlargement, and the complete right bundle branch block [18,20]. The abnormal electrocardiogram findings in athletes are the following: abnormal T-wave inversion, ST-segment depression, complete left bundle branch block, profound non-specific intra-ventricular conduction delay, ventricular pre-excitation, abnormal QT prolongation, Brugada type 1 pattern, profound sinus bradycardia or first degree, high-grade atrioventricular block, multiple premature ventricular contractions, atrial tachyarrhythmias, and multiple premature ventricular contractions [18,19,21].

Regarding the FQRST angle, only a few studies have analyzed its changes correlated with exercise. For example, in elite skiers, better athletic performance is associated with a narrower QRS-T angle [22]. In contrast, other authors highlighted that the FQRST is wider in healthy athletes compared to healthy normal subjects, regardless of the use of protein supplements [23]. Also, the potential use of FQRST for sudden death risk stratification in athletes was proposed [24].

However, it is less established whether these changes occur during physical exercise or are only residual in the post-exercise period as chronic adaptations. Also, in healthy people, the spectrum of FQRST changes, in correlation with the amount of physical activity performed, is not well defined. Considering the inconsistency of the data presented, our research focused on testing the difference in FQRST between groups of individuals with different physical activity levels (PAL).

The main objective of the research was to identify the influence of PAL in FQRST in healthy young adults. The secondary objectives were to measure and describe the relationship between FQRST and some variables (sex, nutritional status, and other ECG parameters) as an explanatory approach.

## 2. Materials and Methods

### 2.1. Aim of the Study and Participants

We conducted an observational cross-sectional study on a sample of healthy young adults (*n* = 124, mean age 20.28 ± 2.23 years, 71 men and 53 women, age range 18–27 years). Before the data gathering, we obtained written informed consent from each subject to participate in the study, according to the ethical principle of research with humans. The study received approval from the Research Ethics Committee of the Research Center for Promoting Excellence in Professional Training, University of Pitesti (reference number 146/7 March 2022).

Eligible participants were recruited based on the following criteria: young adults (age range 18–27 years), good health status confirmed by a medical professional, acceptance for medical data recording, and completing a physical activity assessment questionnaire. The subjects’ exclusion criteria were: sports performance practice, history of recent acute or chronic diseases, medication intake during the last week, and pregnancy for women. Sports performance practice was defined as the regular practice of a sport, at the elite level, during the last year. This criterion was used to avoid the interference of left ventricular hypertrophy, specific for sports performance, in the FQRST analysis.

### 2.2. Data Acquisition

The assessments were carried out between 8 am and 12 am, based on the same protocol for each participant: measuring the height (H), determining the body weight (W), recording the ECG at rest, and then administering a questionnaire to evaluate the physical activity of the last seven days. Starting from W (in kg) and H (in m), we calculated the body mass index (BMI):

(1) $$\mathrm{BMI}\;=\;\frac{W}{H^{2}}$$

The Cardimax FX-7402 device, Fukuda Denshi Co. Ltd. (Tokyo, Japan), with 12 derivations, was used for ECG recording. We recorded the ECG signals for all participants in the supine position, for a minimum of 30 s, with a speed of 25 mm/s and a sensitivity of 5 mm/mV. Then, we manually determined the heart rate (HR), measured the QT interval, and calculated the FQRSA, FTA, and FQRST. For the measurement of the QT interval, we used the tangent method in lead II [25]. Then, with the Bazett formula, we calculated the corrected QT interval (QTc) [26]:

(2) $$\mathrm{QTc}\;=\;\frac{\mathrm{QT}}{\sqrt{\mathrm{RR}}}$$

For QTc interpretation, according to most experts, the normal values taken into consideration were QTc < 440 ms for adult males and <460 ms for adult females [27].

For the FQRSA and FTA manual measurements, we used the leads I and aVF. On the hexaxial reference system, we determined the areas of positive and negative deflections of the QRS complex and T wave [1]. According to previous literature, normal FQRS was in the range of −30° to 90°, and a normal FTA was between −15° to 105° [4]. Then we calculated the FQRST by using the following formula [28]:

(3) $$\mathrm{FQRST}\;=\;\pm \;\arctan\;\frac{2*\mathrm{aVF}}{\sqrt{3}*I}$$

The formula expresses the angle between the mean QRS and T wave vectors, determined in leads I and aVF. For interpretation of FQRST, an angle below 90° was considered normal, and values ≥ 100° abnormal [1,3,13].

Another objective for the subjects’ assessment concerned their PAL. For this purpose, we used the International Physical Activity Questionnaire—Short Form (IPAQ). IPAQ short form is an instrument with fair to moderate validity, designed primarily for population surveillance of PAL among adults (age range of 15–69 years) [29]. IPAQ assesses physical activity during the last seven days in four main domains: leisure, domestic and gardening (yard), work, and transport. The IPAQ considers three specific types of physical activity: walking, moderate-intensity, and vigorous-intensity activities [30].

From IPAQ, we obtained categorical and continuous indicators of physical activity. The IPAQ scoring protocol allows us to compute IPAQ scores expressed in MET-minutes/week based on weighting each type of activity by its energy requirements. Computation of the IPAQ scores requires the summation of the duration (in minutes) and frequency (days) of the mentioned activities. The collected data with IPAQ, as a self-report measure, categorize individuals into three classes of physical activity: 1 = low PAL, 2 = moderate PAL, and 3 = high PAL [31].

### 2.3. Outcomes and Statistical Analysis

This study provided the following variables: age, sex, W, H, BMI, IPAQ score (MET-minutes/week), category of PAL, HR, QT and QTc intervals, FQRSA, FTA, and FQRST. Data analysis was carried out using the software IBM SPSS 20.0 (IBM Corp., Armonk, NY, USA) [32]. The raincloud plot was plotted by https://www.bioinformatics.com.cn/en (accessed on 19 January 2023), a free online platform for data analysis and visualization [33]. Descriptive statistics are shown as mean (SD) or as median (25th–75th percentile) for continuous variables and as count (%) for categorical variables. Means (SD) were chosen for continuous variables when the normality hypothesis was rejected (*p* < 0.05), as assessed with the Shapiro-Wilk test. The FQRST variable is further described in relation to the PAL level using raincloud plots. Normality hypothesis was not rejected for W, QT, QTc, and FTA, for a level of significance for rejecting the null of 0.05. We applied nonparametric tests for inferential statistical analysis: the Kruskal-Wallis H test and the Mann–Whitney U-test (with effect size calculation using Cohen’s r) for independent large samples (*n* > 20) to check the statistical significance of the differences between groups. The Kruskal-Wallis test was used to address the main objective of the study, with the null hypothesis stating that the medians of the independent samples are equal, at a significance level of 0.05. We conducted the Mann-Whitney test as a post-hoc analysis and corrected the considered *p*-values of statistical significance of 0.05 using the Bonferroni correction method. Groups were initially defined according to PAL and then to sex. Another analysis considered the average heart rate in the groups of subjects with different PAL. The same methodology was utilized to examine differences in FQRST based on sex levels and to investigate differences in heart rate based on PAL levels.

We also realized a correlational analysis of data based the Spearman’s rank-order correlation. The variables considered for the correlational analysis were age, W, H, BMI, HR, QT, QTc, FQRSA, FTA, and FQRST. Initially, we tested the bivariate normality between the analyzed variables with a normal distribution (W, QT, QTc, and FTA) required for parametric correlational analysis, but we rejected it. Consecutively, we applied the Spearman analysis for all pairs of variables. Our goal was to understand the relationship between all the recorded variables, including FQRST, as part of a comprehensive analysis. Finally, a linear regression analysis was run to determine the relationship between pair variables that are significantly correlated and are appropriate to the objective of the research (independent variable BMI, dependent variable FQRST).

## 3. Results

The main results are reported in Table 1 as baseline descriptive statistical indicators. For the statistical analysis of data, we divided the sample according to PAL into three subgroups (with low PAL, moderate PAL, and high PAL), and then according to sex into two subgroups (men and women).

From the baseline characteristics of the subjects, regarding their nutritional status, we noticed an average healthy weight range (mean BMI = 22.04 ± 2.87), according to the NIH/WHO guidelines for BMI [34]. In the sample, 3.2% of the participants were underweight, 81.5% had normal weight status, 14.5% had overweight, and 0.8% had obesity class I.

From the ECG records, all subjects had a normal sinus rhythm without significant conduction or repolarization abnormalities. The QT interval varied from 280 to 442 ms (mean QT = 362.28 ± 30.47 ms), for the corresponding HR at rest ranging from 48 to 120 beats/min (mean HR = 72.52 ± 13.67 bpm). The corrected QTc fluctuated in the group of participants between 309 and 465 ms (mean QTc = 394.89 ± 32.40 ms). The QTc intervals were within normal limits for 94.4% of the subjects and prolonged for 5.6% of them, according to the sex reference values [27].

Analysis of FQRSA data revealed normal values for 94.4% of the subjects and a right deviation for 5.6% of cases. Instead, the FTA was in the normal range for each participant. Finally, the FQRST, as a composite variable, was normal in the participants’ group (below 100°).

Another investigated parameter was the PAL of the subjects. The analysis of the IPAQ scores showed that 28.2% of the participants had a low PAL, 41.1% had a moderate PAL, and 39.7% had a high PAL.

We started the inferential statistical analysis with a rank-based nonparametric test (the Kruskal-Wallis H test) to compare the differences between data series with a nonnormal distribution (FQRST). Thus, we applied the mentioned test to understand whether FQRST differed in the sample group based on PAL. The dependent variable was the FQRST, and the independent variable was the PAL, with three independent groups: subjects with low, medium, and high PAL. The results showed a statistically significant difference in FQRST between the different PAL, χ2(2) = 71.92, *p* < 0.001, with a mean rank of FQRST 100.56 for low PAL, 61.19 for moderate PAL and 29.21 for high PAL.

Next, we ran a Mann-Whitney test to compare differences between pairs of two independent groups with the continuous dependent variable FQRST. Independent groups were defined based on their PAL: group 1 with low PAL, group II with moderate PAL, and group 3 with high PAL. The results of this inferential analysis are presented in Table 2, Table 3 and Table 4.

**Figure 1:**
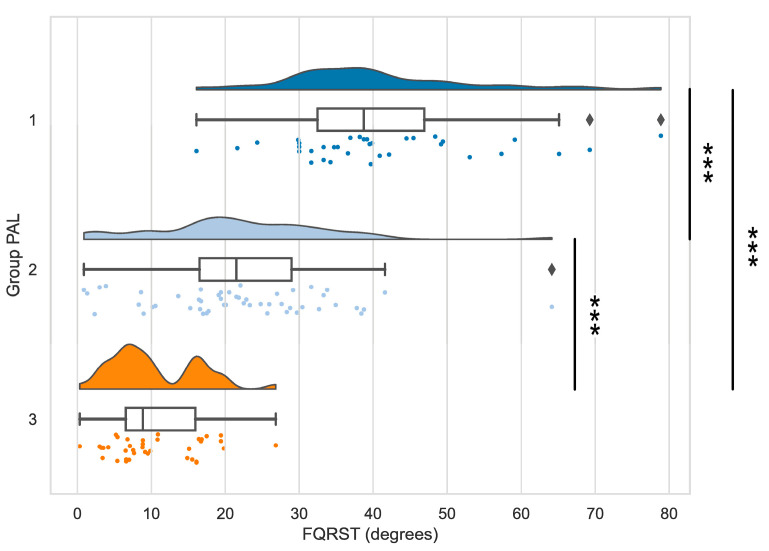
The distributions of FQRST data depending on the PAL level. Note—Stars indicate significant differences (*p* < 0.001) for the comparisons of FQRST between PAL groups 1 and 2, 2 and 3, 1 and 3, respectively (Mann-Whitney tests).

The analysis of the differences between groups revealed statistically significant results for FQRST (*p* < 0.001) when comparing groups 1 and 2, 2 and 3, and, respectively, 1 and 3. In our statistical analysis, we used the value of the Bonferroni-corrected threshold of statistical significance of 0.016. The magnitude of the differences between the data series indicated a large effect size (r > 0.5), according to the r classification system [35]. The raincloud plot of the distributions of FQRST data depending on the PAL level is presented in Figure 1 [33].

The same Mann-Whitney test was also applied to compare differences between men and women, with the continuous dependent variable FQRST. The results of this analysis were not statistically significant (Table 5).

Regarding the variable HR, we reported some differences between the groups of subjects with different PAL without statistical significance. Thus, in group 1 with low PAL, we recorded a mean HR of 75.69 ± 14.14 beats/min, in group 2 with moderate PAL of 73.10 ± 12.82, and in group 3 with high PAL the mean value of 68.84 ± 13.86.

Our data were also analyzed using bivariate Spearman’s correlation r_s_ with statistical significance to understand better the relationship between the recorded variables (Table 6). In Spearman’s correlation matrix, should be noticed the correlation between FQRST and BMI (R = 0.24, *p* < 0.05).

**Figure 2:**
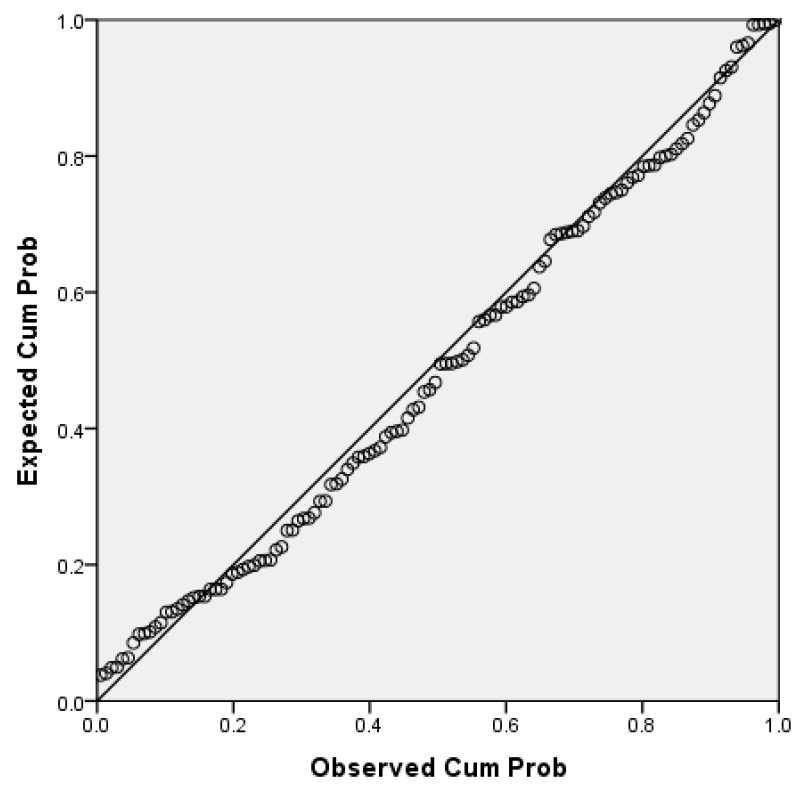
The normal P-P plot of regression standardized residuals (independent variables BMI, dependent variable FQRST.

After the correlation analysis, a linear regression was the next step to link the correlated variables of interest for our research purpose. We chose the BMI for the linear regression analysis with FQRST since BMI is a composite variable that reflects the nutritional status and has intensively been studied and associated with FQRST. While FTA is also correlated with FQRST, it is a variable that is used to calculate FQRST through vector geometry, making the relationship between these variables more complex and less relevant to our objective. We run a simple linear regression analysis to determine the effect of BMI (the independent and predictor variable) on the FQRST (the dependent outcome variable). According to the normal P-P plot of regression, the standardized residuals of data considered were approximately normally distributed, and the required assumption to run a linear regression was assured (Figure 2).

For the mentioned series of data, we didn’t find significant outliers. The assumption of independence of residuals was checked using the Durbin-Watson test for autocorrelation in the residuals, which assesses the serial dependence of data [36]. The obtained value of 1.86 for the Durbin-Watson test offered statistical confidence for the regression performed.

We found a regression model between FQRST and BMI (Table 7) with statistical significance (*p* < 0.001). Therefore, a part of the variability of the FQRST (0.09%) is explained by the nutritional status in terms of BMI.

The following regression equation reports how well the analysis fits the data:

FQRST = −13.42 + BMI × 1.69(4)

## 4. Discussion

This analysis found a statistically significant difference in FQRST between the three groups of subjects, classified by their PAL in low, moderate, and high categories of physical activity (*p* < 0.001). Based on the mean rank differences between groups, our results suggest that the widening of FQRST is present in sedentary young adults, even if the angle values do not exceed the limit of normality. At the same time, the narrowing of this angle is characteristic of individuals with an active lifestyle. It should be noticed that, overall, the study participants had no pathological changes on the ECG recordings, except the presence of a prolonged QTc interval in 5.6% of them and a right deviation of FQRSA for 5.6% of them.

Our findings indicate that the interpretation of the FQRST should consider the PAL of the participants since it offers a new perspective for ECG-data stratification. This study also analyzed the relation between BMI and FQRST and found that nutritional status had a predictable influence on the difference in orientation between ventricular depolarization and repolarization (*p* < 0.001). The relationship between these two variables assumes a moderate correlation (R = 0.30, *p* < 0.001).

The presented data offered the opportunity to discuss the results in light of other similar studies. Thus, we identified only two recent types of research that focused on FQRST changes in elite performance athletes, with somewhat contradictory results [22,23]. One of these studies demonstrated that FQRST is wider in athletes compared to normal healthy participants [23]. Instead, the second-mentioned study revealed that between athletes (skiers) and nonathlete control subjects, no differences related to FQRST were observed [22]. The same authors concluded that higher athletic performance, but not athletic training, is associated with the FQRST’s narrowing [22].

In two other older studies, we found similar opposite findings. Thus, one research identified a mean QRS-T angle larger in athletes and U waves more prominent [37], while another reported a wider QRS-T angle (>60°) present only in 14% of a group of professional football players [38]. In conclusion, there is a lack of studies evaluating the usefulness of FQRST in systematic ECG screening of athletes. Relative evidence suggests its applicability in preventing sudden cardiac death in sports [39].

The explanations for our results are related to the physiologic cardiovascular adaptations, specifically for exercise. Usually, well-conditioned individuals have an increased basal vagal tone [40] and present signs of cardiac remodeling [38,41]. These cardiovascular adaptations to physical activity differ depending on the type of physical solicitation: endurance (aerobic) training and strength (anaerobic) training [41]. The acute responses to endurance exercise reflect the fraction of maximal oxygen uptake that is used [42], while the chronic cardiovascular adaptation to exercise includes increased maximal oxygen uptake [41]. In this last situation, exercise-induced cardiac remodeling occurs over time, with left ventricular hypertrophy (eccentric or concentric) and modifications in both systolic and diastolic functions [43]. This adaptation induces an increase in the systolic flow and better hemodynamic body reactivity during exercise. At the cellular metabolic level, a physiological adaptation to oxidative stress appears that directs the cellular homeostasis towards better regulation of redox homeostasis and signaling [44].

For our study, we considered the long-term adaptation to exercise to explain our findings. The IPAQ assessed the PAL of the subjects related to their participation in repetitive physical activities that may induce adaptive cardiovascular effects. Regarding the vagal component of adaptation to physical effort, we can analyze the average heart rate in the groups of subjects with different PAL. Although the mean HR differences between the groups are low and without statistical significance, the tendency is to decrease the HR in the most active people. This trend respects the concept of increased vagal tone in actively trained people.

In this study, we provided a comprehensive assessment of the FQRSA, FTA, and FQRST in the group of participants. If normal intervals are provided as a guideline for FQRSA and FTA, in the case of FQRST, only the normal upper limit is considered in clinical practice. Usually, the direction of the resultant vector of ventricular depolarization is directly opposite to the ventricular repolarization one, producing a narrow FQRST [3]. Since FQRST quantifies the planar electrical ventricular gradient [45], a very wide QRS-T angle may indicate dysregulation of ventricular repolarization [3,5].

In our study, all subjects had normal FQRST (under 100°), but the lower values were specific to those with high PAL. As a novelty of our study, we demonstrated that PAL influences the FQRST values, even if they were in the normal range. Thus, optimal values of FQRST appear in physically active young adults, while in sedentary individuals the angle tends to enlarge without reaching the abnormal level.

As a result, we can estimate that early intervention to increase the PAL in individuals may protect future body dynamics in terms of FQRST values. This explanation is plausible since FQRST reflects the disjunction between ventricular depolarization and repolarization and has a direct relationship with the risk of developing cardiovascular impairments, such as sudden death, cardiac ischemia, or ventricular arrhythmia [46].

Another discussion concerns the consequences of widening the QRS-T angle on hemodynamics. Thus, a widened spatial QRS-T angle may represent poor ventricular stretch [47]. A negative relationship has been established in the context of physical activity (during and immediately after exercise) between spatial QRS-T angle and diastolic blood pressure caused by poor ventricular stretch [47]. On the other hand, a large FQRST may be associated with left ventricular hypertrophy. Left ventricular hypertrophy is specific in sports performance [48]. For this reason, we excluded from the study athletes with high levels of training.

The final result of our study highlighted the regression model that demonstrated a significant and positive relationship between FQRST and BMI. Previous research suggested a positive weak correlation of FQRST with BMI [49]. Some authors explained the association of these parameters on young adults (20–30 years of age) by the effect of adiposity on the chest electric properties that influence the recording of the surface ECG signals [2]. The impact is more pronounced in lean or obese patients, but supplementary clinical investigations are needed to clarify this phenomenon. Other possible interference in the relation between FQRST and BMI is explained by the different levels of athletic training and physical activity of the subjects [2]. As we already have mentioned, such factors might affect the values of the QRS-T angle due to the electrophysiological and geometric changes specific to the syndrome of athlete’s heart [50].

Our results are also in accord with those of other authors who investigated the FQRST by referring to the World Health Organization classification for BMI [50]. Through a multivariate linear regression analysis, this research showed that BMI is an independent determinant of the QRS-T angle [51], and the mean values obtained for the FQRST are similar to ours. Investigating the nutritional factor associated with other cardiovascular risk factors, an association between metabolic syndrome and its components, with spatial QRS-T angle, both in men and women, was determined [52].

It is interesting that among all of the constitutional variables analyzed in association with FQRST (W, H, BMI), the highest correlation was achieved with BMI (R = 0.30) and then with H (R = −0.21). Another research stated that H is the most significant constitutional determinant of ECG since an increase in H determines the augmentation of most QRS and ST-T parameters [53].

To conclude, the use of advanced ECG parameters, such as the FQRST, gets new values for the interpretation of the complex factors that interfere with the individual health status. Beyond the controversies in the literature, the data presented here confirm the applicability of FQRST for understanding the body’s adaptations to exercise in healthy young adults. Taking into account the constitutional parameters in various clinical situations, the ECG diagnosis and the stratification of risk factors for health can be improved. Additional research on large samples and with interventional design is needed to refine these findings.

This study has several limitations. Firstly, the validity of the IPAQ collected data depends on the honesty and subjectivity of the respondents. Then, the statistical analysis was carried out through non-parametric tests of the ranks, which may lack power compared to parametric tests. For the results’ interpretation, we did not consider other categorical variables and concomitant cardiovascular risk factors of the subjects, such as genetic predisposition, smoking, alcohol consumption, type of diet, etc. Also, for the analysis of the ECG data, we eliminated the cases of left ventricular hypertrophy, which could influence the analyzed variables. However, we tried to minimize this inconvenience by excluding performance athletes from among the subjects, who usually present these ECG changes. Another limitation of the study is that we did not conduct a statistical power analysis to determine the sample size. While we considered other factors such as similar studies in the literature to expect having an appropriate sample size, it remains possible that our study lacked of statistical power for the analyses we conducted.

## 5. Conclusions

The physical activity regime of young adults significantly influences the concordance between ventricular depolarization and repolarization, reflected in the FQRST’s width. A narrow FQRST is associated with a high PAL in healthy young adults, while in sedentary subjects, the angle tends to increase without exceeding the normal upper limit. We also identified a significant regression model between the BMI and the FQRST.

## Figures and Tables

**Table 1:** Baseline descriptive statistical indicators for investigated parameters.

Variable	Entire Sample (*n* = 124)	Group with Low PAL (*n* = 35)	Group with Moderate PAL (*n* = 51)	Group with High PAL (*n* = 38)	Men (*n* = 71)	Women (*n* = 53)
Age (years)						
Percentile						
25th	19	19	19	19	19	19
50th (median)	19.50	20	19	20	20	19
75th	21	22	20	22	21	20
W (kg)						
Mean	64.85	66.23	62.78	66.34	70.15	57.74
SD	10.80	10.86	9.78	11.83	9.52	7.99
H (cm)						
Percentile						
25th	163	159	163	168.75	174	158
50th (median)	172	167	172	177	177	162
75th	178.75	174	178	179.25	180	169
BMI (kg/m^2^)						
Percentile						
25th	20.01	20.90	19.95	19.44	20.20	19.31
50th (median)	21.31	23.53	20.39	21.22	21.71	20.55
75th	24.01	26.49	22.76	22.08	24.38	23.48
HR (beats/min)						
Percentile						
25th	62	63	63	58.75	60	68
50th (median)	71	73	73	68	68	75
75th	81	86	82	75.75	80	83
QT (ms)						
Mean	362.28	359.83	359.31	368.53	359.41	366.13
SD	30.47	30.37	26.02	35.59	33.05	26.47
QTc (ms)						
Mean	394.89	400.51	394.29	390.50	385	408.13
SD	32.40	27.60	36.97	29.82	34.02	24.70
FQRSA (degrees)						
Percentile						
25th	52.27	68.95	53.90	37.36	52.07	52.32
50th (median)	68.02	78.97	67.83	57.49	68.21	67.83
75th	77.36	67.83	76.10	68.51	77.78	77.06
FTA (degrees)						
Mean	43.84	38.64	46.51	45.04	45.78	41.24
SD	14.19	11.53	13.61	16.13	15.73	11.45
FQRST (degrees)						
Percentile						
25th	9.94	31.64	16.44	6.25	8.60	16.10
50th (median)	20.31	38.74	21.50	8.85	19.11	24.32
75th	33.66	48.40	29.17	16.10	31.63	35.66
IPAQ score(MET-minutes/week)						
Percentile						
25th	500.25	348	690	1650	518	480.50
50th (median)	975	444	970	1770	1116	690
75th	1610	490	1116	1878	1756	1376.50
Distribution of PAL						
count and %	124 (100%)	35 (28.23%)	51 (41.13%)	38 (30.64%)	71 (57.26%)	53 (42.74%)

Note—W: weight; H: height; BMI: body mass index; HR: heart rate; QT: electrical interval QT; QTc: corrected electrical interval QT; FQRSA: frontal QRS axis; FTA: frontal T axis; FQRST: frontal QRS-T angle; IPAQ: International Physical Activity Questionnaire; SD: standard deviation; PAL: physical activity level; *n*: group size.

**Table 2:** Comparison of recorded parameters (Mann Whitney test) between group 1 (*n* = 35, with low PAL) and group 2 (*n* = 51, with moderate PAL), grouping variable IPAQ category.

Variable	Group 1 (Mean Rank)	Group 2 (Mean Rank)	U	Z	*p*	r
FQRST	62.87	30.21	214.5	−5.96	<0.001	1.68

Note—*n*: number of subjects; PAL: physical activity level; IPAQ: International Physical Activity Questionnaire; FQRST: frontal QRS-T angle; U: Mann–Whitney’s U-test values, Z: scores; *p*: thresholds of statistical significance; r: Cohen’s effect size coefficient.

**Table 3:** Comparison of recorded parameters (Mann Whitney test) between group 2 (*n* = 51, with moderate PAL) and group 3 (*n* = 38, with high PAL), grouping variable IPAQ category.

Variable	Group 2 (Mean Rank)	Group 3 (Mean Rank)	U	Z	*p*	r
FQRST	56.98	28.92	358	−5.06	<0.001	1.27

Note—*n*: number of subjects; PAL: physical activity level; IPAQ: International Physical Activity Questionnaire; FQRST: frontal QRS-T angle; U: Mann–Whitney’s U-test values, Z: scores; *p*: thresholds of statistical significance; r: Cohen’s effect size coefficient.

**Table 4:** Comparison of recorded parameters (Mann Whitney test) between group 1 (*n* = 35, with low PAL) and group 3 (*n* = 38, with high PAL), grouping variable IPAQ category.

Variable	Group 1 (Mean Rank)	Group 3 (Mean Rank)	U	Z	*p*	r
FQRST	55.69	19.79	11	−7.22	< 0.001	3.16

Note—*n*: number of subjects; PAL: physical activity level; IPAQ: International Physical Activity Questionnaire; FQRST: frontal QRS-T angle; U: Mann–Whitney’s U-test values, Z: scores; *p*: thresholds of statistical significance; r: Cohen’s effect size coefficient.

**Table 5:** Comparison of recorded parameters (Mann–Whitney test) between the group of men (*n* = 71) and the group of women (*n* = 53), grouping variable IPAQ category.

Variable	Group Men (Mean Rank)	Group Women (Mean Rank)	U	Z	*p*	r
FQRST	58.24	68.21	1579	−1.53	0.127	0.28

Note—*n*: number of subjects; FQRST: frontal QRS-T angle; U: Mann–Whitney’s U-test values, Z: scores; *p*: thresholds of statistical significance; r: Cohen’s effect size coefficient.

**Table 6:** Correlation output (Spearman’s correlation coefficient r_s_) between the recorded variables and the statistical significance level “*p*” (*n* = 124).

Variable	Age	W	H	BMI	HR	QT	QTc	FQRSA	FTA	FQRST
age	1.00									
W	0.50 *	1.00								
H	0.31 *	0.68 *	1.00							
BMI	0.32 *	0.70 *	0.09	1.00						
HR	−0.19 *	−0.09	−0.32 *	0.12	1.00					
QT	0.08	0.02	0.14	−0.03	−0.46 *	1.00				
QTc	−0.15	−0.09	−0.25 *	0.14	0.56 *	0.41 *	1.00			
FQRSA	−0.06	−0.14	−0.13	−0.07	−0.05	−0.12	−0.15	1.00		
FTA	−0.27	−0.09	0.05	−0.21 *	−0.07	−0.10	−0.12	0.29 *	1.00	
FQRST	0.07	−0.03	−0.22	0.24 *	0.12	−0.10	0.02	0.57 *	−0.27 *	1.00

Note—W: weight; H: height; BMI: body mass index; HR: heart rate; QT: electrical interval QT; QTc: corrected electrical interval QT; FQRSA: frontal QRS axis; FTA: frontal T axis; FQRST: frontal QRS-T angle; *: *p* < 0.05 was considered statistically significant (2-tailed); *n*: group size.

**Table 7:** Model summary, ANOVA report, and coefficients for simple linear regression analysis—BMI versus FQRST (*n* = 124).

Variable	R	R Square	Adjusted R Square	SE	F	*p*	β0	SE	*p*	95%LB	95%UB	β1	SE	*p*	95%LB	95%UB
**FQRST**	0.30	0.09	0.084	15.30	12.33	< 0.001	−13.42	10.68	<0.001	−34.56	7.72	1.69	0.48	<0.001	0.74	2.64

Note—BMI: body mass index; FQRST: frontal QRS-T angle; R: Pearson’s coefficient of correlation; SE: standard error; F: test for overall significance for the linear model; *p*: level of statistical significance; β0: the intercept coefficient; β1: the regression coefficient; 95%LB and 95%UB: lower bound and upper bound of the 95% confidence interval; *n*: group size.

## Data Availability

The data are available on request from the corresponding author. All data relevant to the study are included in the article.
